# Cardiogenic shock in a patient with combined severe aortic and mitral regurgitation treated by a totally percutaneous approach: a case report

**DOI:** 10.1093/ehjcr/ytaf185

**Published:** 2025-04-15

**Authors:** Francesco Lo Iudice, Amelia Ravera, Alfonso Campanile, Stefano Romei, Francesco Vigorito

**Affiliations:** Cardiology Department, S. Giovanni Di Dio E Ruggi D’Aragona Hospital of Salerno, Largo Città di Ippocrate, Salerno 84131, Italy; Cardiology Department, S. Giovanni Di Dio E Ruggi D’Aragona Hospital of Salerno, Largo Città di Ippocrate, Salerno 84131, Italy; Cardiology Department, S. Giovanni Di Dio E Ruggi D’Aragona Hospital of Salerno, Largo Città di Ippocrate, Salerno 84131, Italy; Cardiology Department, S. Giovanni Di Dio E Ruggi D’Aragona Hospital of Salerno, Largo Città di Ippocrate, Salerno 84131, Italy; Cardiology Department, S. Giovanni Di Dio E Ruggi D’Aragona Hospital of Salerno, Largo Città di Ippocrate, Salerno 84131, Italy

**Keywords:** Cardiogenic shock, Mitral regurgitation, Aortic regurgitation, TAVI, Impella, Transcatheter edge-to-edge repair, Case report

## Abstract

**Background:**

Combined severe aortic regurgitation and severe mitral regurgitation is a condition associated with high mortality, where evidence, about proper management, is still scarce, especially in critical clinical conditions such as cardiogenic shock.

**Case summary:**

An 86-year-old female with severe aortic and mitral regurgitation was admitted due to acute pulmonary oedema, rapidly deteriorating in cardiogenic shock refractory to medical treatment. Haemodynamic stabilization was achieved only after implantation of an Impella CP, through a trans-femoral approach. Considering the prohibitive surgical risk, the mitral valve regurgitation was treated with a transcatheter edge-to-edge repair procedure, which allowed to successfully wean the patient from Impella. Subsequently, a transcatheter aortic valve implantation was performed. The patient’s clinical status improved to such a level that a rehabilitation program was successfully implemented.

**Discussion:**

Our report shows that an entirely percutaneous approach, to manage a combined severe aortic and mitral regurgitation, complicated by cardiogenic shock, is feasible and effective.

**Learning points**

- Coexistence of severe aortic regurgitation with severe functional mitral regurgitation is associated with a bad prognosis and can lead to clinical and haemodynamics deterioration up to cardiogenic shock.
- Left ventricular unloading using Impella CP is an effective treatment in cardiogenic shock due to left-sided valve regurgitation.
- A totally percutaneous approach using mitral transcatheter edge-to-edge repair and TAVI is feasible in high-risk patients with combined severe aortic and mitral regurgitation.

## Introduction

Multiple valvular heart disease is a condition where evidence is still poor and specific recommendations are lacking.^1,2^ Transcatheter interventions represent a valid option for patients at high surgical risk and their successful use in critical conditions, such as cardiogenic shock has been recently reported.^3^

## Summary figure

**Figure:**
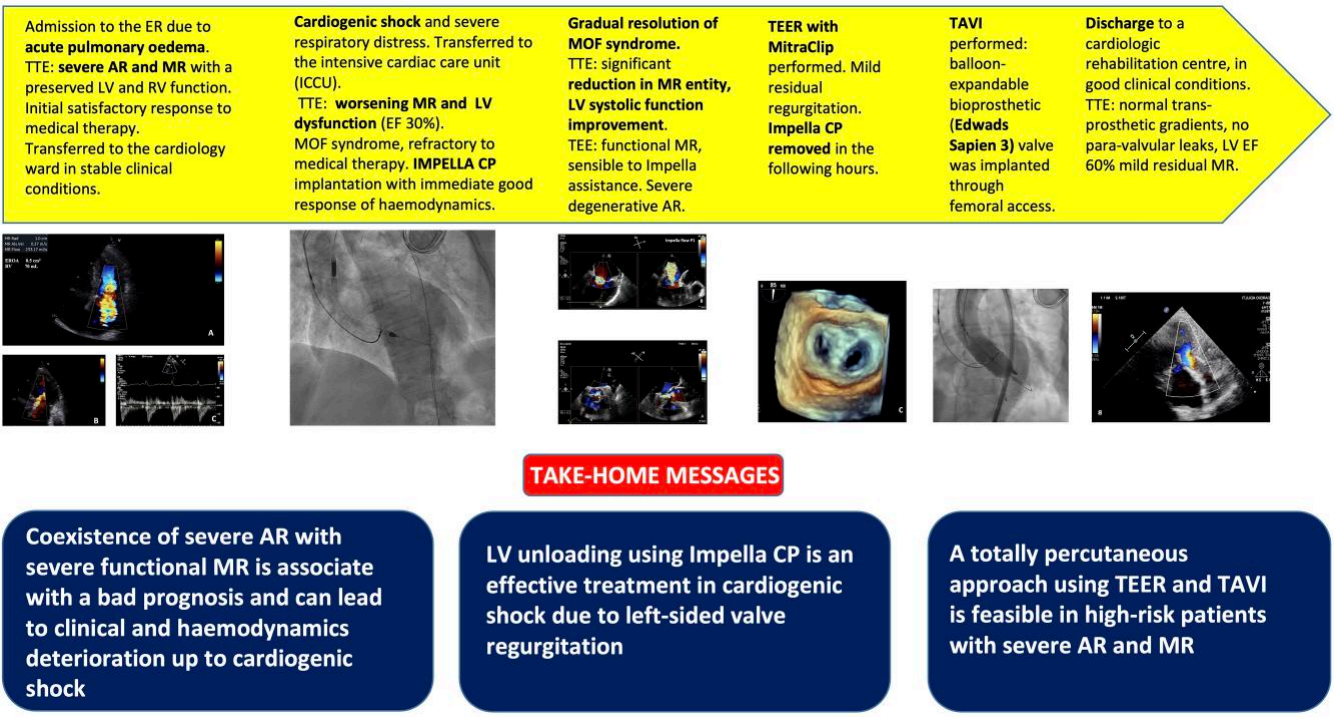


## Case presentation

An 86-year-old female presented to the emergency department because of dyspnea at rest. The patient background was characterized by a history of moderate degenerative aortic regurgitation (AR) and moderate mitral regurgitation (MR), dual chamber pacemaker implantation for sick-sinus syndrome, paroxysmal atrial fibrillation, arterial hypertension, and hypercholesterolaemia.

On admission, the patient was orthopneic, with an oxygen saturation of 87% in room air, and an arterial blood pressure of 85/50 mmHg. On physical examination, she had bilateral pulmonary crackles, an apical systolic murmur, and a diastolic murmur in the aortic focus.

**Figure 1:**
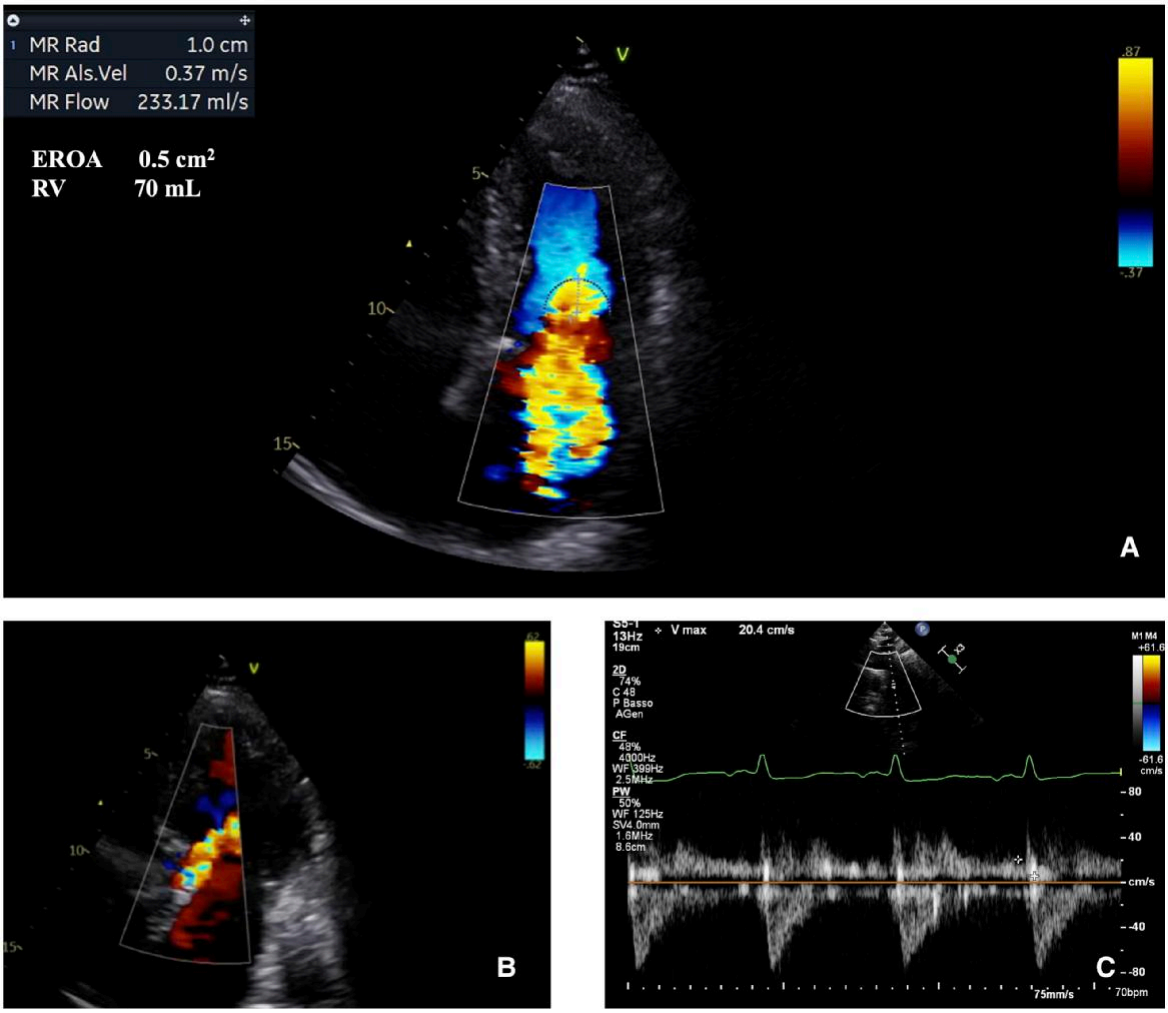
TTE on admission. (*A*) Evaluation of MR effective orifice area (EROA) and regurgitant volume (RV) using PISA method. (*B*) Color-Doppler evaluation of AR. (*C*) Pulsed-wave Doppler showing significant diastolic flow reverse in descending aorta.

The electrocardiogram showed an atrial and ventricular paced rhythm with a heart rate of 60 bpm. Chest X-ray was consistent with pulmonary oedema. Laboratory data are summarized in Supplementary material online, *Table S1*. Transthoracic echocardiography (TTE) showed severe AR (EROA 0.4 cm^2^; RV 80 mL; PHT 190 ms), severe MR (EROA 0.5 cm^2^; RV 70 mL; systolic reversal flow in pulmonary veins), a mild left ventricular (LV) dilatation with an ejection fraction (EF) of 50% (see Supplementary material online, *Video S1*; *Figure 1*). Right ventricle (RV) was not dilated with a normal systolic function (TAPSE 21 mm). A moderate tricuspid regurgitation was detected, with an estimated systolic pulmonary arterial pressure of 50 mmHg. The inferior vena cava was dilated with a reduced inspiratory collapse.

**Figure 2:**
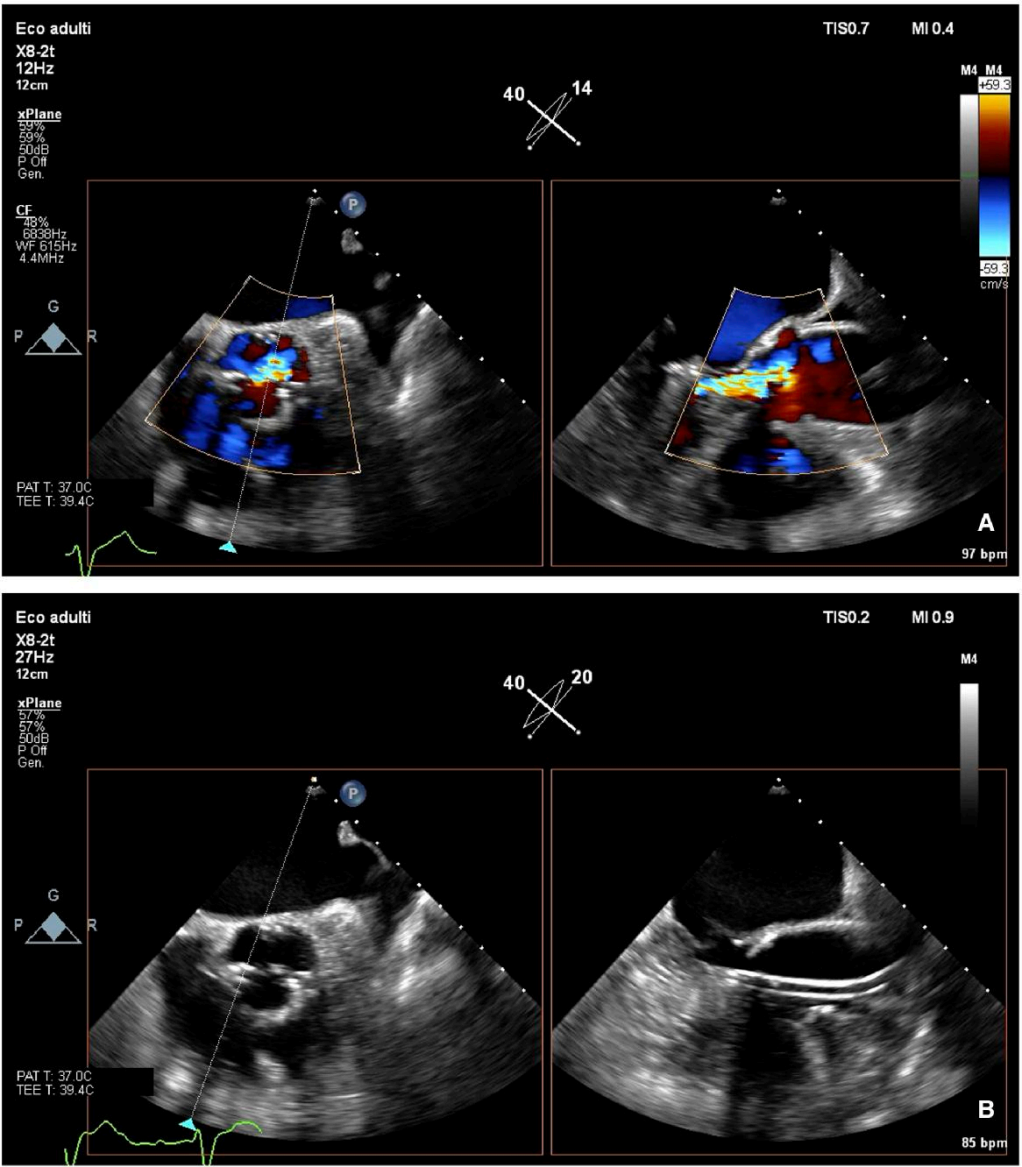
TEE evaluation of aortic valve. (*A*) Severe aortic regurgitation (central jet) in a tricuspid valve with calcification of commissures. (*B*) Impella CP catheter laying in the commissure between right and non–coronary cusp, without significant interaction with cusps closure.

**Figure 3:**
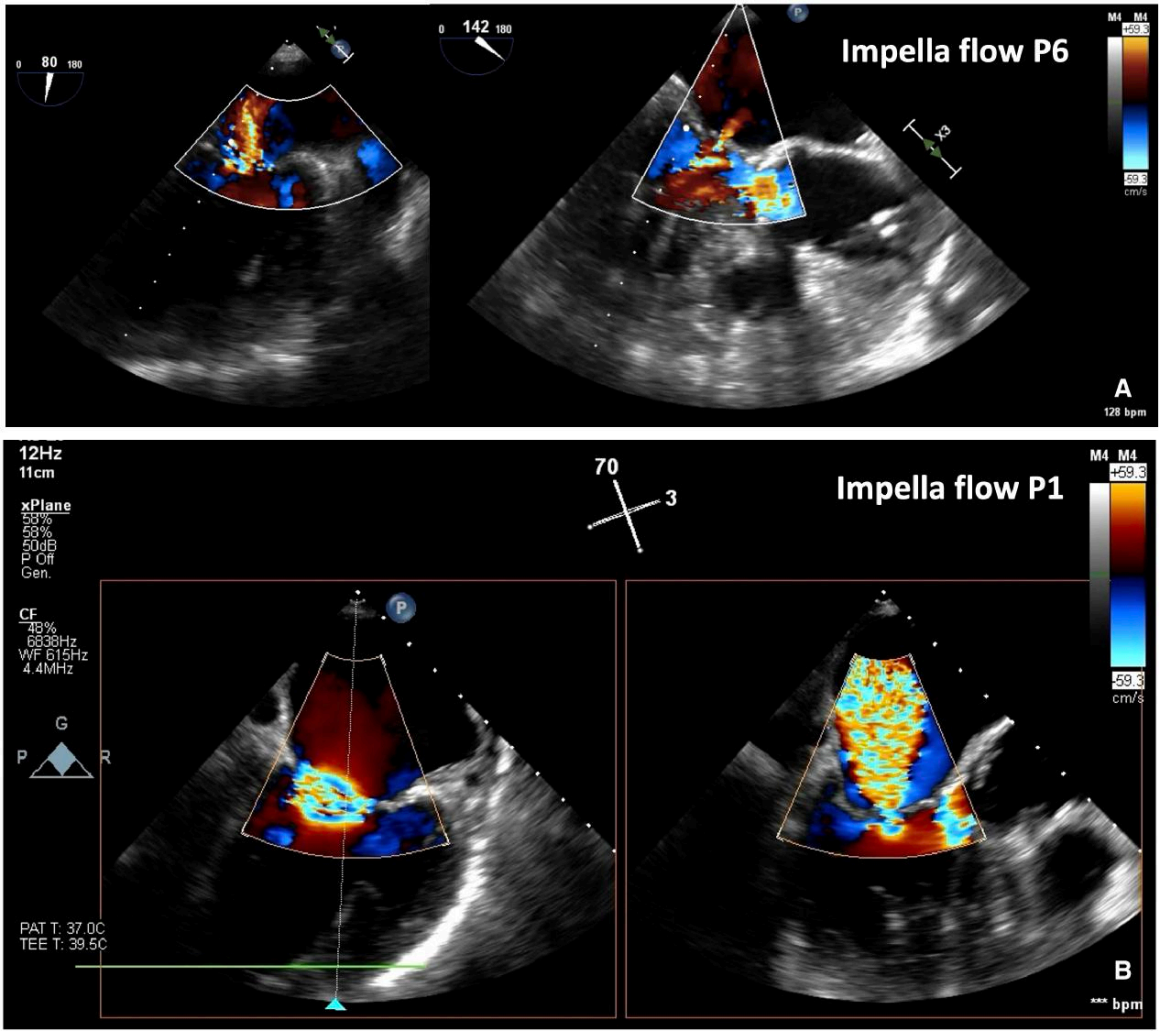
TEE evaluation of MR. (*A*) Impella CP flow level P6: moderate MR. (*B*) Impella CP flow level P1: severe MR.

**Figure 4:**
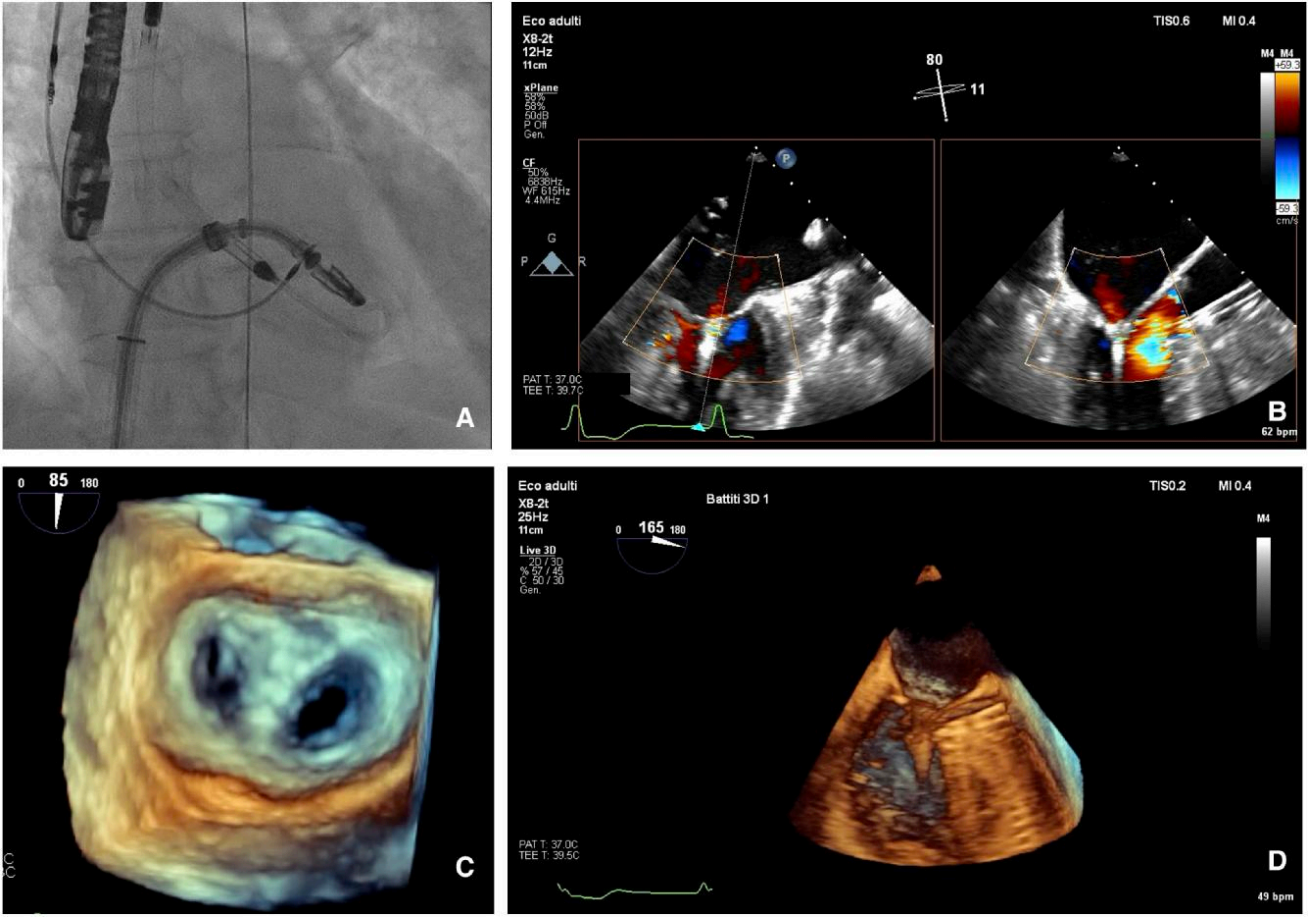
TEER procedure. (*A*) Fluoroscopy showing MitraClip system together with Impella CP in LV. (*B*) TEE showing mild residual MR after MitraClip placement. (*C*) 3D echocardiography of mitral valve after MitraClip implantation. (*D*) 3D echocardiography showing MitraClip and Impella CP catheter in close proximity without interference.

**Figure 5:**
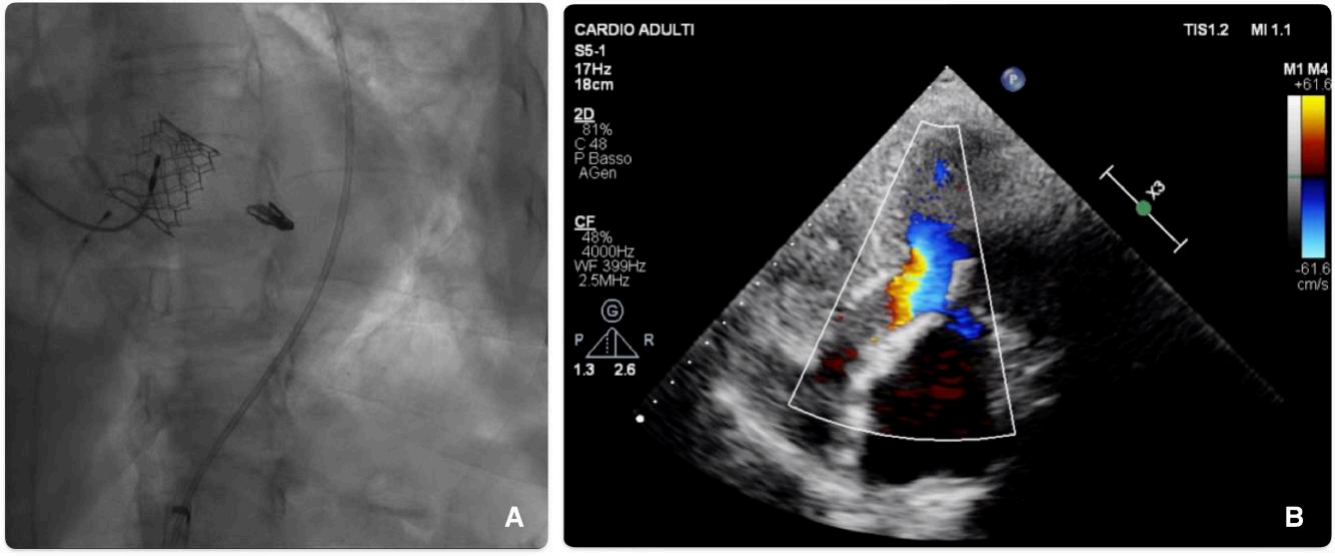
(*A*) Fluoroscopy showing Sapien 3 prosthesis in aortic position and MitraClip. (*B*) TTE after TAVI and TEER.

The patient was treated with oxygen support, low-dose norepinephrine, and high-dose furosemide infusion. The initial patient response was satisfactory, and she was transferred to the cardiology ward in stable clinical conditions. Nevertheless, soon the patient became refractory to diuretics and after 48 h she was again in severe respiratory distress, hypotensive, with a rapid increase of the lactate levels (12 mmol/L). An urgent admission to the Intensive Cardiac Care Unit was required. Non-invasive ventilation, inotropic, and vasopressor support were started. TTE showed a significant worsening of MR with a prominent coaptation gap (5 mm) between leaflets; LV systolic function was depressed (EF 30%, with diffuse hypokinesia; estimated anterograde stroke volume index 15 mL/mq; estimated cardiac index 1.05 L/m^2^). RV function was still preserved and estimated systolic pulmonary arterial pressure was 60 mmHg. Laboratory findings showed a picture of multi-organ failure (see Supplementary material online, *Table S1*). The patient was refractory to the medical treatment and, following a heart team discussion, the decision for a temporary mechanical cardiac support with IMPELLA CP® (Abiomed) was established. IMPELLA CP® is a microaxial pump draining blood from LV and ejecting it into ascending aorta, therefore providing unloading of LV and support to cardiac output (up to 4.3 L/min). It can be set to 9 levels, from P1 to P9, corresponding to increasing rotational velocity of the turbine and, accordingly, increasing levels of anterograde flow and LV unloading. After the implantation, through right femoral artery, the patient had a prompt and dramatic improvement (blood pressure 110/60 mmHg, satisfactory urine output, and reduction of dyspnoea). TTE immediately after implantation revealed a significant reduction in MR entity that was still severe, but without an evident coaptation gap between leaflets. LV systolic function gradually improved, no worsening of AR for the interaction of the catheter with cusps was observed. Estimated systolic pulmonary arterial pressure was 30 mmHg. After weaning the patient from inotropic and vasopressor support, a transesophageal echocardiography (TEE) was performed, with evidence of severe degenerative AR (*Figure 2*; Supplementary material online, *Video S2*) with a wide central jet (vena contracta width 9 mm) in a tricuspid valve with normal systolic leaflets opening, marked cusps fibrosis, and nodular calcification of commissures. Aortic root and ascending aorta were not significantly dilated (respectively, 36 and 35 mm) and sino-tubular junction was preserved (26 mm). Functional moderate MR (biplane vena contracta 0.45 cm) was detected during LV assistance with Impella CP® at P6 flow level, which increased to severe (biplane vena contracta 0.95 cm) when preload was increased by reducing, for a very short time interval, LV unloading at flow-level P1, the lowest allowed by the device (*Figure 3*; Supplementary material online, *Video S3*). Central jet originating from A2-P2 was evident; leaflets were fibrotic with normal length, preserved diastolic motion, and systolic apical tethering; anulus was dilated (antero-posterior diameter 36 mm, inter-commissural diameter 38 mm). The surgical risk was judged prohibitive (EUROSCORE 22.7%). Therefore, a total percutaneous strategy was planned. An attempt to wean the patient from Impella assistance failed due to haemodynamic and clinical destabilization; therefore, we decided to treat mitral valve for first, keeping Impella on site during the procedure. The patient was judged suitable for transcatheter edge-to-edge repair (TEER) with MitraClip® (Abbot) system and G4 XTW clip was chosen due to favourable anatomical features (posterior leaflet length was 12 mm, planimetric mitral valve area was 4.5 cm^2^, anterograde pressure gradient 2 mmHg, trans-septal puncture height was 46 mm). Consequently, one clip was implanted between A2 and P2 (*Figure 4*; Supplementary material online, *Video S4*). Residual regurgitation was trivial and anterograde transvalvular mean pressure gradient was 5 mmHg. Impella CP was removed in the following hours. The patient remained haemodynamically stable and asymptomatic at rest. Nevertheless, AR, as expected, remained severe (see Supplementary material online, *Video S5*). Therefore, a cardiac computed tomography (CT) scan, in order to plan transcatheter aortic valve implantation (TAVI), was performed: aortic annulus area was 469 mm^2^, cusps were not calcified. Coronary CT evaluation did not show significant atherosclerotic stenosis. Subsequently, through right arterial femoral access, an Edwards Sapien 3, 26 mm bioprosthesis, was implanted in aortic position. Considering the absence of calcification on aortic cusps, a slight over-inflation of the balloon (2 mL) compared with the recommended value was performed to promote anchoring of the prosthesis. Final procedural aortography showed correct position of the prosthesis and absence of significant residual regurgitation (see Supplementary material online, *Video S6*), haemodynamic parameters remained stable and an increase in diastolic blood pressure (from 45 to 70 mmHg) was observed after prosthesis implantation. Patient remained stable in the following days. Echocardiogram showed normal trans-prosthetic gradients (mean gradient of 9 mmHg), the absence of para-valvular leaks, improvement of LV systolic function (EF 60%) while residual MR remained mild (*Figure 5*). Clinical conditions gradually improved and, after 1 week, the patient was transferred to a cardiologic rehabilitation centre.

At 6-month follow-up, the patient was alive and in good clinical condition. No sign of pulmonary nor peripheral congestion were detected at physical examination. Echocardiography confirmed the good outcome of the procedures on mitral and aortic valve.

## Discussion

To the best of our knowledge, only one case has been published in the literature showing the feasibility of a total percutaneous management of AR and MR complicated by cardiogenic shock.^4^ However, our report is still unique, due to the complex approach performed, characterized by stabilization of haemodynamics through the implantation of a mechanical cardiac support (IMPELLA CP ®) in the first place.

Coexistence of significant (at least moderate) MR was found in 14% of patients with at least moderate AR in a retrospective study: MR mechanism was functional in the majority (64%) of cases, and patients with significant AR plus significant functional MR showed the worst survival rate.^5^ In our patient, recent worsening of degenerative AR, from moderate to severe, was accompanied by marked worsening of MR, due to a functional mechanism (annular dilatation and leaflet tethering). Increasing volume overload and reduction of forward stroke volume put into crisis compensation mechanism of LV, which eventually became dysfunctional leading to severe cardiogenic shock, refractory to medical therapy. Monitoring of haemodynamic parameters was crucial for guiding the management of cardiogenic shock: invasive monitoring of arterial blood pressure was performed, while central venous pressure and pulmonary arterial pressure were indirectly monitored non-invasively with daily echocardiographic evaluation.

In this scenario, LV mechanical assistance was deemed necessary, and benefits of LV unloading through Impella CP overcame the risk of AR worsening due to the interaction of the catheter with aortic leaflets. Indeed, successful use of Impella CP® in acute AR-driven cardiogenic shock has been previously reported.^6^ In our case, haemodynamic and clinical improvement with ventricular unloading was rapid and dramatic. After stabilization of the patient, we decided first to treat MR, despite its functional etiology suggested a possible improvement if aortic valve was treated for first.^7^ The severity of the clinical picture imposed us a different approach: considering that the patient was dependent on Impella assistance (an attempt to wean the patient from Impella had failed) we decided to begin with TEER of mitral valve, which, unlike TAVI, allowed us to keep Impella on site during the procedure. Indeed, during haemodynamic deterioration, evidence of marked worsening of functional MR had been detected through echocardiography and, therefore, we were confident that treating MR would prevent an early new decompensation after Impella removal. Correction of functional MR, indeed, allowed successful weaning from Impella, keeping haemodynamic stability, so we were able to safely perform cardiacCT for TAVI planning. There is growing experience of TEER in acute clinical conditions, even during Impella assistance.^8^ TAVI is currently approved for treatment of aortic stenosis, though AR represents an ‘off-label’ indication. Nevertheless, there evidence of good feasibility and safety of TAVI in AR patients and its use in this condition is spreading.^9^

## Conclusions

An entirely percutaneous treatment of AR and MR complicated by cardiogenic shock is a feasible option in high-risk patients.

## Supplementary Material

ytaf185_Supplementary_Data

## Data Availability

The data underlying this article are available in the article and in its online Supplementary material.

## Lead author biography

Dr Francesco Lo Iudice received his medical degree from Federico II University of Naples, where he also held his residency in Cardiology. During his residency, he also completed a fellowship in structural imaging and interventional echocardiography at S. Raffaele Hospital of Milan. He is currently working as intensive care cardiologist and cardiac imaging consultant at S. Giovanni di Dio e Ruggi d’Aragona Hospital of Salerno.

## Notes

**Consent:** The authors confirm that written consent for submission and publication of this case report including images and associated text, in accordance to the COPE guidelines, has been obtained from the patient.

**Funding:** Open access was supported by Abiomed Europe GmbH.
