# Supplementary material for: Cardiogenic shock in a patient with combined severe aortic and mitral regurgitation treated by a totally percutaneous approach: a case report
Source: Eur Heart J Case Rep. 2025 Apr 15;9(4):ytaf185. doi: 10.1093/ehjcr/ytaf185 (PMC12032388; doi:10.1093/ehjcr/ytaf185)
Supplement: ytaf185_Supplementary_Data [file ytaf185_supplementary_data.zip › Table S1 revised.pdf]

Table S1 Main laboratory values during the whole patient admission period

	Admission to ER	Admission to ICCU (MOF syndrome)	Discharge
eGFR (ml/min)	38	19	93
BUN (mg/dL)	132	275	27
Creatinine(mg/dL)	1.29	2.5	0.41
GOT (U/l)	59	1900	19
GPT (U/l)	98	1024	21
CPK (U/l)	113	858	22
Tn (ng/L)	27	22228	7
WBC (10^3/µL)	13.73	21.97	5.23
Hb (g/dL)	11.8	9.5	10.8
PLT (10^3/µL)	147	198	178
PT-INR	1.04	1.99	1.08
PCT (ng/mL)	0.38	0.8	0.11
Lactate (mmol/L)	1.3	12.1	0.6
pH	7.37	7.08	7.36
$PaCO_2$ (mmHg)	32	64	45
PaO_2 (mmHg)	72	55	94
HCO_3^- (mEq/L)	29	37.4	24
FiO_2 (%)	40	50	21
P/F Ratio	180	110	447
BNP (pg/mL)	1116	2578	123

BNP: brain natriuretic peptide; BUN: blood urea nitrogen; CPK: creatine phosphokinase; eGFR: estimated glomerular filtration rate (MDRD formula); ER: emergency room; FiO2: Fraction of inspired O2; GOT: glutamic oxaloacetic transaminase; GPT: glutamic pyruvic transaminase; Hb: hemoglobin; ICCU: intensive cardiac care unit; MOF: multi-organ failure; PCT: procalcitonine; PLT: platelets; P/F: PaO2/FiO2 Ratio; PT-INR: prothrombin time international normalized ratio; Tn: I troponin (high sensitivity); WBC: white blood cells.
